# Hatching egg polyunsaturated fatty acids and the broiler chick

**DOI:** 10.1186/s40104-022-00757-5

**Published:** 2022-09-18

**Authors:** Gita Cherian

**Affiliations:** Department of Animal and Rangeland Sciences, 112 Withycombe Hall, Oregon State University, Corvallis, Oregon, 97331 USA

**Keywords:** Chick, Egg, Fatty acid molecular species, Phospholipids

## Abstract

Transgenerational effects of certain nutrients such as essential fatty acids are gaining increased attention in the field of human medicine and animal sciences as a new tool to improve health and animal performance during perinatal life. Omega-3 (n-3) and omega-6 (n-6) fatty acids are denoted by the position of the first double bond from methyl end of the hydrocarbon chain. Alpha-linolenic acid (18:3 n-3) and linoleic acid (18:2 n-6) are essential n-3 and n-6 fatty acids and cannot be synthesized by the vertebrates including chickens. Alpha-linolenic acid and linoleic acid are the parent fatty acids of long chain (> 20–22C) n-3 and n-6 polyunsaturated fatty acids (PUFA) such as eicosapentaenoic acid (20:5 n-3, EPA), docosapentaenoic acid (22:5 n-3/or 22:5 n-6, DPA), docosahexaenoic acid (22:6 n-3, DHA) and arachidonic acid (20:4 n-6). As components of cell membrane phospholipids, PUFA serves as precursors of eicosanoids, act as ligands for membrane receptors and transcription factors that regulate gene expression and are pivotal for normal chick growth and development. Considering the role of egg lipids as the sole source of essential fatty acids to the hatchling, dietary deficiencies or inadequate in ovo supply may have repercussions in tissue PUFA incorporation, lipid metabolism, chick growth and development during pre and early post-hatch period. This review focus on studies showing how maternal dietary n-3 or n-6 fatty acids can lead to remodeling of long chain n-3 and n-6 PUFA in the hatching egg and progeny chick tissue phospholipid molecular species and its impact on chick growth and PUFA metabolism during early life.

## Introduction

Chicken embryonic development takes place in a semi-closed system, the egg, where only exchange of gas and water take place. Hatching egg serves as a reservoir of nutrients prepackaged from maternal (breeder hen) sources. Lipids in egg are synthesized de novo in the hen liver and are present exclusively in the yolk. Information on hatching egg lipids is limited to fat content and fatty acid composition. As the developing broiler chick relies on egg nutrients for over one third of its life span [1], comprehensive lipid profiling in hatching egg may provide insight on relationship between egg lipid composition and its development-related functional properties during pre and early post hatch life.

### Hatching egg lipidome

**Fig. 1:**
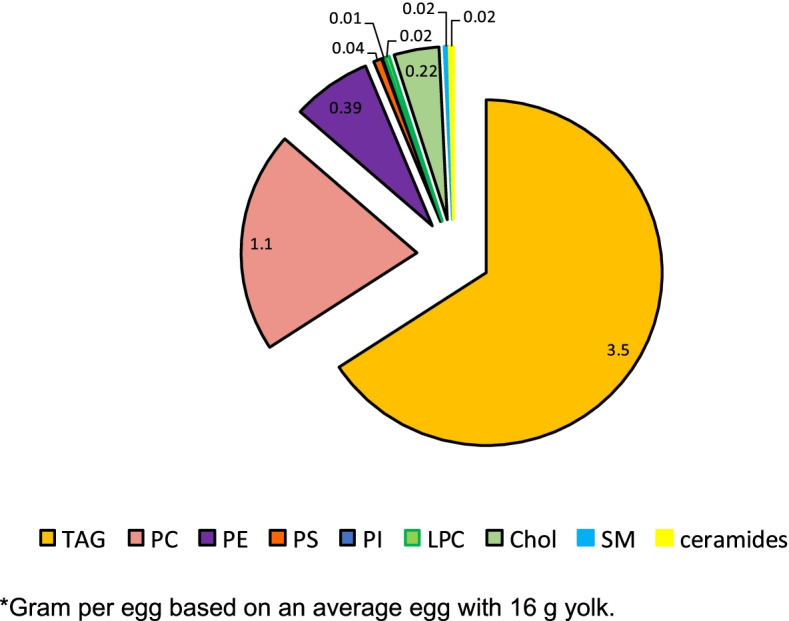
Lipid components in egg^*^. ^*^Gram per egg based on an average egg with 16 g yolk. TAG = Triacylglycerol, PC = phosphatidylcholine; PE = phosphatidylethanolamine, PS = phosphatidylserine; PI = phosphatidylinositol; LPC = lysophosphatidylcholine, Chol = cholesterol; SM = sphingomyelin

Lipidomics is the analysis of lipids on the systems-level and is a subset of metabolome dealing with quantitative phenotyping of small lipid molecules in different lipid classes [2]. Egg lipid is a complex matrix and is comprised of diverse classes of lipids such as triacylglycerol, different glycerophospholipids, ceramides, sphingolipids, and sterols (Fig. 1). Lipid components in egg are involved in diverse metabolic (e.g. energetic, thermogenesis) and physiological (e.g. cellular structural, signaling) functions during avian embryogenesis [1, 3, 4] (Table 1). The composition of the egg lipidome (i.e., the structure and abundance of different lipid classes) and metabolites can vary significantly between different lipid classes [5, 6] and can be influenced by hen age and diet [7–9]. Information on lipidomic characterization is limited to table eggs enriched with n-3 fatty acids produced for human consumption [9–11] with limited information on hatching eggs.

**Table 1:** Lipid components in eggs and their physiological roles during embryogenesis

Neutral lipids (e.g. Triacylglycerol)	Energy metabolism, signal transduction, and function as precursors to other glycerophospholipids
Phospholipids^*^	Structural component of cell membranes Serving as enzyme substrates for lipoprotein metabolism Serving as ligands for receptors, Signal transduction, Supplier of polyunsaturated fatty acids that serves as precursors of different lipid mediators and essential biomolecule. Anti-inflammatory and antiproliferative properties Intracellular traffickers of cholesterol
Sterols	Sterols such as cholesterol and cholesterol ester are involved in lipoprotein assembly and transport of yolk lipids to embryonic tissues.

^*^Phospholipids include: phosphatidylcholine, phosphatidylethanolamine, phosphatidylserine, phosphatidylinositol, phosphatidylglycerol, phosphatidic acid, lysophosphatidylcholine, lysophosphatidylethanolamine, and sphingomyelin

Fatty acids are yolk lipid components and constitutes over 4 g in an average egg and are distributed in the carbon 1 (stereospecific numbering (sn), sn-1), carbon 2 (sn-2), or carbon 3 (sn-3) of the glycerol backbone in triacylglycerol. In phospholipids, the head group is typically esterified at the sn-3 position of the glycerol backbone with the fatty acyl chains attached at the sn*-*l and sn*-*2 positions. The diverse array of alkyl chains containing different combinations of fatty acids in the sn-1, sn-2 (e.g. phospholipids) and sn-3 positions (triglyceride) of the glycerol backbone is referred to as fatty acid molecular speciation. The diversity in positional distribution of fatty acids in the glycerol backbone has significant influence in biological systems with respect to their biophysiological, biochemical properties, structural functions and cellular signaling [12]. Information regarding the positional distribution of fatty acids has limited our understanding in the metabolic roles of PUFA during avian embryogenesis and early chick life. In this context, a lipidomic approach to investigate the role of maternal dietary n-3 or n-6 fatty acids in remodeling n-3 and n-6 PUFA molecular species with emphasis on phospholipids in the hatching egg and its consequence on the broiler hatchling is discussed in this review.

### Egg phospholipids: a reservoir of polyunsaturated fatty acids

Glycerophospholipids or phospholipids, are important biochemical components and constitute ~ 27–28% of total lipids in eggs. Phospholipids contain a variety of polar head groups anchored to the glycerol backbone (sphingosine backbone in the case of sphingomyelin). A list of different phospholipids that are present in the hatching egg is shown in Fig. 1. Among the different phospholipids, > 70% (~ 1.3–1.4 g/average egg) is phosphatidylcholine (PC) and over 25% (~ 0.25 g/average egg) is phosphatidylethanolamine (PE) with the remaining being other minor phospholipids (e.g. phosphatidylserine, phosphatidylinositol, lysophosphatidyl choline and lysophosphatidyl ethanolamine) [13]. The functional properties of egg phospholipids during avian embryogenesis is shown in Table 1. As reservoirs of long chain PUFA such as arachidonic acid, EPA and DHA, that are anchored at the sn-2 position of glycerol backbone, egg phospholipids play pivotal roles as building blocks of the biological membranes in embryonic tissues, enzymatic pathways, lipid and eicosanoid metabolism [14–17]. Additionally, metabolites derived from PUFA degradation are important intracellular signaling molecules involved in processes such as proliferation and apoptosis [4, 16, 18]. The role of hen’s diet in modulating phospholipid fatty acid composition of eggs has been well documented [14, 19] with limited information on fatty acid molecular species orientation. In order to unravel biochemical mechanisms and to understand the role(s) of phospholipids during embryogenesis it is essential to identify and quantify individual phospholipid lipid classes, their content and molecular species orientation. Detailed information on different phospholipid classes and positional distribution of fatty acids in hatching eggs will contribute to our understanding of role of maternal diet and PUFA metabolism during embryogenesis and its impact on chick health and growth. In view of the importance of essential n-3 fatty acids in early chick development [1, 14, 17, 20], series of studies were conducted in our laboratory using breeder hen diet, fatty acid modified egg and the neonatal chick as a model to explore in ovo nutrition and lipid metabolism during avian embryogenesis.

**Fig. 2:**
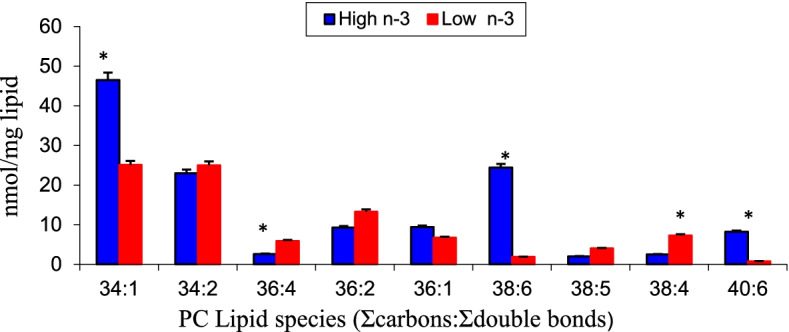
Changes in fatty acid molecular species of egg yolk phosphatidylcholine as affected by maternal dietary high n-3 or low n-3 fatty acid diet. High n-3 and Low n-3 represent breeder hen diet containing 3.5% fish oil or sunflower oil as the source of long chain n-3 fatty acid or n-6 fatty acid (linoleic acid). *Significantly different (*P* < 0.05). *n* = 6

To investigate the effect of maternal diet on yolk phospholipid composition and molecular speciation, eggs were collected from breeder hens fed diets high or low in n-3 fatty acids. Fish oil or sunflower was used as the source of long chain n-3 fatty acid (EPA, DHA) or n-6 fatty acid (linoleic acid) in the hen diet to produce High n-3 or Low n-3 eggs. The oils were added to the hen diet at 3.5% and the hens were fed a 2866 kcal, and 16.0% CP diets [21]. Total long chain n-3 fatty acids (EPA + DPA + DHA) in the egg constituted 12% and 1.5% for High n-3 and Low n-3 eggs, respectively. The arachidonic content of eggs was 1.3% and 4.6% for High n-3 and Low n-3 eggs, respectively [22]. The total n-6 to n-3 fatty acid ratio in the egg was 15.0:0.08. This ratio was created to simulate the n-6:n-3 fatty acid ratio in commercial hatching eggs that are high in n-6 fatty acids with negligible n-3 fatty acid content [22]. Characterization and quantification of different phospholipid molecular species with respect to precise esterified fatty acyl groups and their amounts were conducted using electrospray ionization mass spectrometry (ESI-MS). Details of the ESI-MS methodology are reported earlier [8]. Mapping of the PC molecular species revealed that 34:2 (16:0/18:2) (sum of hydrocarbons:sum of unsaturation) and 34:1(16:0/18:1) constituted the major (~ 50%) species of yolk PC. Some of the major molecular species of egg yolk PC are shown in Fig. 2. The effect of hen’s diet was very prominent in PC 34:1 (16:0/18:1), 38:6 (16:0/22:6), 38:4 (18:0/20:4) and 40:6 (18:0/22:6). The longer chain omega-6 (arachidonic acid) and omega-3 PUFA (DHA) were located mainly in the sn-2 position of egg PC. High n-3 fatty acid diet resulted in a significant increase in phospholipid species containing DHA (16:0:/22:6), (18:0/22:6) with a concomitant reduction in arachidonic acid species such as 36:4 (16:0/20:4), and 38:4 (18:0/20:4). Total PC (mol%) was higher in High n-3 compared to Low n-3 eggs. PC being the predominant phospholipid class present in chicken eggs that serves as reservoir of PUFA, inadequate supply of PC may limit PUFA supply to the developing chick embryo. These results demonstrate that maternal dietary n-3 or n-6 fatty acid alters phospholipid composition and fatty acid molecular species orientation in egg yolk. The most notable effect from the supplementation of High n-3 diet is the marked increase of DHA-containing 38:6 and 40:6 phospholipid species, which occurred mainly at the expense of 36:4 and 38:4 particularly arachidonic acid in egg PC. Similar responses to dietary marine algal biomass rich in long chain omega-3 PUFA supplementation in laying hens have been reported for DHA accumulation in egg yolk PC and PE [10, 11]. These results on characterization of egg yolk PC n-3 and n-6 PUFA species elucidates how maternal diet alters the orientation of n-3 and n-6 PUFA molecular species in the egg phospholipid classes. Since phospholipids especially PC serve as the major building blocks of the chick embryo cell membranes, quantification and identification of the molecular species is essential for a complete understanding of the role of in ovo exposure to PUFA on lipid metabolism in the progeny chicks.

### Polyunsaturated fatty acid remodeling in chick tissue during early growth

**Fig. 3:**
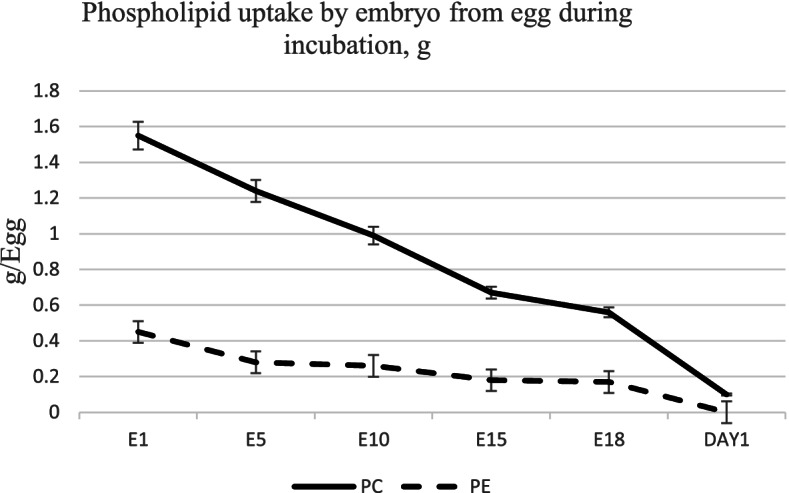
Pattern of phosphatidylcholine and phosphatidylethanolamine transfer from hatching egg to the chick embryo during incubation. PC = phosphatidylcholine, PE = phosphatidylethanolamine, E = embryonic age. The weight of PC and PE in in egg yolk or remnant yolk sac (g) from day one of incubation through hatching period. *n* = 8

**Fig. 4:**
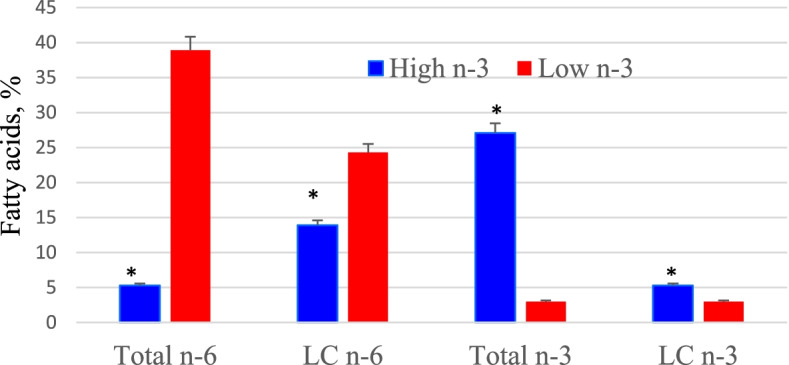
Fatty acid composition of newly hatched chick cardiac tissue from eggs high or low in n-3 fatty acids. High n-3 and Low n-3 represent breeder hen diet containing 3.5% fish oil or sunflower oil as the source of long chain n-3 fatty acid or n-6 fatty acid (linoleic acid). LC = long chain (> 20C). *Significantly different (*P* < 0.05). *n* = 6

The rapidly growing chick embryo needs phospholipid-rich PUFA for membrane biogenesis and growth and this is evidenced by the uptake of PC and PE from hatching egg to the embryo. By the end of 21-day incubation, over 90% of PC and PE are transferred from the egg to the developing chick embryo (Fig. 3). Therefore, availability of long chain n-3 and n-6 PUFA from egg yolk phospholipids are crucial for embryonic growth and chick tissue maturation. A substantial body of evidence exists regarding the role of maternal dietary fatty acids in modulating embryo tissue PUFA composition [20, 23–25]. However, very little is known about the impact of maternal diet and egg n-3 PUFA composition on specific molecular species on hatched chick tissue phospholipids. Previous research from author’s lab reported significant impact of maternal dietary conjugated linoleic acid in remodeling phospholipid molecular species in the developing embryo and altering chick growth and hatchability [8]. It is likely that alterations in hatching egg phospholipid composition and molecular species orientation will impact hatched chick tissue phospholipid PUFA orientation as well as other cellular functions. In this context, maternal diet and egg PUFA composition as a target for remodeling phospholipid molecular species composition and PUFA metabolism of the progeny cardiac tissue in the hatched chick was investigated. Cardiac tissue of the newly hatched chick was taken as a model to assess PUFA molecular species orientation because our previous studies has shown maternal dietary n-3 and n-6 PUFA composition can significantly alter PUFA composition and eicosanoid generation in the cardiac tissue of the newly hatched chicks [21, 26]. High n-3 or Low n-3 eggs were produced (diet details discussed earlier) and were incubated and the cardiac tissue of the hatched chick was isolated for PUFA analysis. Over 9-fold and 1.7-fold increase in total and long chain n-3 fatty acids were observed in the cardiac tissue of High n-3 chicks when compared to Low n-3 chicks (Fig. 4). Notably, this increase in n-3 PUFA incorporation was associated with a significant decrease in proinflammatory eicosanoid (e.g. prostaglandin E_2_) production by the cardiac tissue of High n-3 chicks attesting to the role of egg PUFA in altering lipoid metabolism in the hatchling [21].

**Fig. 5:**
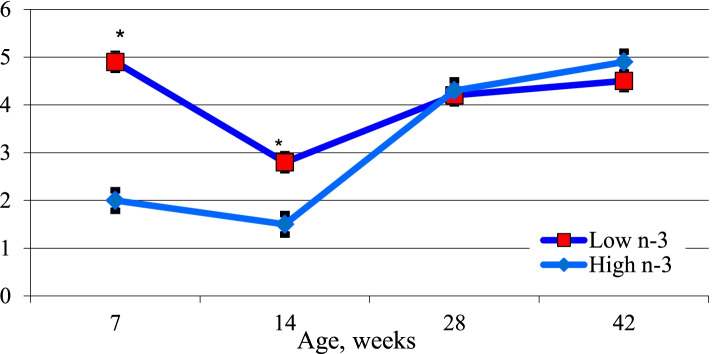
Post hatch changes in arachidonic acid content in the cardiac ventricle (mg/100 g) of progeny chicks as affected by maternal diet. High n-3 and Low n-3 represent breeder hen diet containing 3.5% fish oil or sunflower oil as the source of long chain n-3 fatty acid or n-6 fatty acid (linoleic acid). Chicks were fed a diet devoid of long chain n-3 or n-6 fatty acids during the 42-day feeding trial. *Significantly different (*P* < 0.05). *n* = 6

**Fig. 6:**
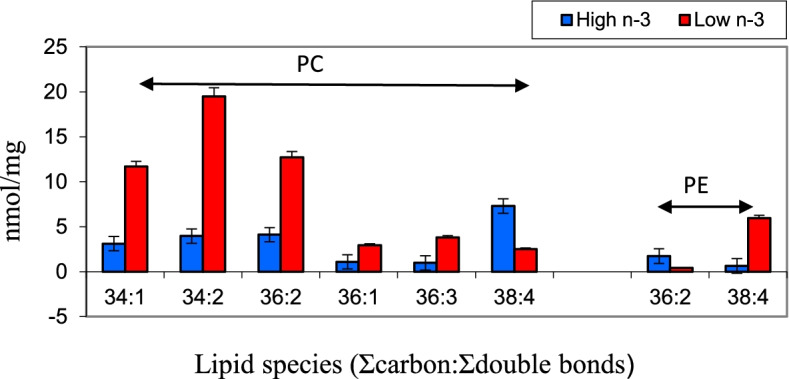
Post hatch changes in the cardiac tissue phospholipid molecular species in the cardiac tissue of progeny chicks at day 14 of age as affected by maternal diet. High n-3 and Low n-3 represent breeder hen diet containing 3.5% fish oil or sunflower oil as the source of long chain n-3 fatty acid or n-6 fatty acid (linoleic acid). PC = phosphatidylcholine; PE = phosphatidylethanolamine. Hatched chicks were fed a diet devoid of long chain n-3 or n-6 fatty acids during the 42-day feeding trial. All bars significantly different (*P* < 0.05). *n* = 6

**Fig. 7:**
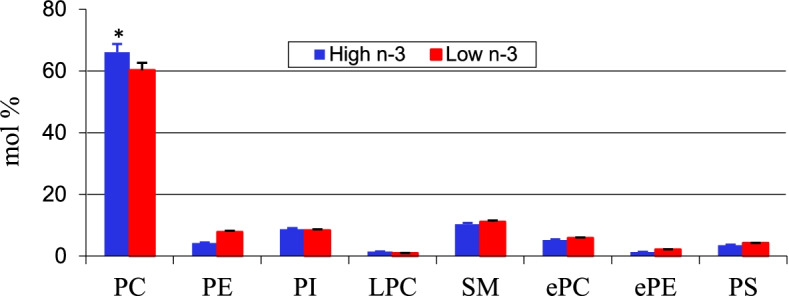
Post hatch changes in the cardiac tissue phospholipid classes of progeny chicks at day 14 of age as affected by maternal diet. High n-3 and Low n-3 represent breeder hen diet (2800 kcal and 16.5% CP) containing 3.5% fish oil or sunflower oil as the source of long chain n-3 fatty acid or n-6 fatty acid (linoleic acid). PC = phosphatidylcholine; PE = phosphatidylethanolamine, PS = phosphatidylserine; PI = phosphatidylinositol; LPC = lysophosphatidylcholine SM = sphingomyelin. Hatched chicks were fed a diet devoid of long chain n-3 or n-6 fatty acids during the 42-day feeding trial. *Significantly different (*P* < 0.05). *n* = 6

The long-term effect of maternal diet and egg PUFA composition on cardiac ventricle PUFA profile was tested in progeny chicks. Chicks hatched from High n-3 and Low n-3 eggs were fed a diet lacking in long chain n-3 or n-6 fatty acids to simulate the current diet fed to modern-day broilers. Fatty acid composition of the cardiac ventricle was analyzed at different points during post hatch growth (d 7, 14, 28, 42). The results revealed a significant increase in arachidonic acid in the cardiac tissue of Low n-3 chicks. The effect of maternal diet remained up to d 14 in progeny chicks with respect to arachidonic acid content (Fig. 5). A similar response to maternal dietary PUFA on progeny tissue retention of fatty acids are reported earlier [14, 20, 27]. Mapping of PC and PE molecular species of cardiac ventricle of chicks done on day 14 of growth revealed a significant increase in n-3 fatty acid species in the PC and PE of chicks hatched from High n-3 eggs (Fig. 6). Highest difference was found in PC 34:2 (16:0/18:2) species where a 3.9-fold increase was observed in Low n-3 chicks when compared to High n-3 chicks. The 38:4 species (18:0/20:4) in PE was over 9-fold higher in Low n-3 chicks comparison with High n-3 chicks. In addition to this, a significant increase in the content of PC in cardiac ventricle of High n-3 chicks were observed which was evident up to day 14 of post hatch growth (Fig. 7). These significant changes in cardiac phospholipids attests to the role of early diet in modulating lipid metabolism in progeny chicks.

Arachidonic acid, EPA, and DHA are esterified at the sn-2 position of cardiac tissue phospholipids and after release by phospholipase A2 are further metabolized by cyclooxygenases and lipooxygenase to different eicosanoids that are mediators of inflammation. Eicosanoids derived from arachidonic acids are more proinflammatory than those derived from n-3 fatty acids [16, 18]. For instance, the selective positioning of fatty acids in the sn-2 position, particularly arachidonic acid vs. DHA, in Low n-3 chicks provides an intact source of these fatty acids for the generation of proinflammatory lipid mediators in progeny chicks as evidenced by our reported studies [26, 28]. In the cardiovascular system, arachidonic acid-derived eicosanoids are responsible for smooth muscle constriction, platelet aggregation, and decreasing thrombus formation [29, 30]. The reduction in proinflammatory eicosanoids observed in the chicks from High n-3 eggs may be due to higher availability of precursor long chain n-3 fatty acids in cell membrane phospholipids. Changes to cardiac ventricle phospholipid molecular species orientation may alter membrane biophysical and biochemical properties highlighting the importance of early exposure to n-3 PUFA through in ovo sources. Taken together, these results extended the understanding in phospholipid composition, n-3 PUFA enrichment patterns in progeny chick cardiac tissue, and eicosanoid generation in newly hatched chick through maternal diet and egg n-3 PUFA manipulation.

The period immediately after hatch is the most critical period in the life of a broiler chicken contributing to mortality and culls. In addition, cardiovascular disorders associated with acute heart failure (sudden death syndrome) and chronic heart failure (ascites, cyanosis, hypoxemia) cause welfare concerns and economic losses to the poultry industry world-wide [31, 32]. In otherwise normal broiler flocks, taken together, early death and metabolic and cardiovascular disorders contribute to the major economic loss at the farm level [31–33]. The cardiac lipids of broiler birds fed a commercial broiler diet may contain 12% to 15% of fatty acids as arachidonic acid, due to the predominance of corn and n-6 fatty acid-rich current diet, hence the predominant bioactive long chain PUFA in the cardiac tissue [14, 27]. Therefore, the role of maternal diet and egg PUFA as a target for controlling inflammatory and cardiac diseases in meat-type broiler chickens needs to be investigated in detail. Furthermore, diets high in n-6 fatty acids are considered an important epigenetic factor contributing to increased incidence of diseases involving inflammatory and coronary vascular diseases in humans [34, 35]. Thus, this information may advance the knowledge in the role of early diet and the etiology of lipid-related developmental origin of diseases.

### Maternal dietary lipids and post hatch chick growth performance

**Fig. 8:**
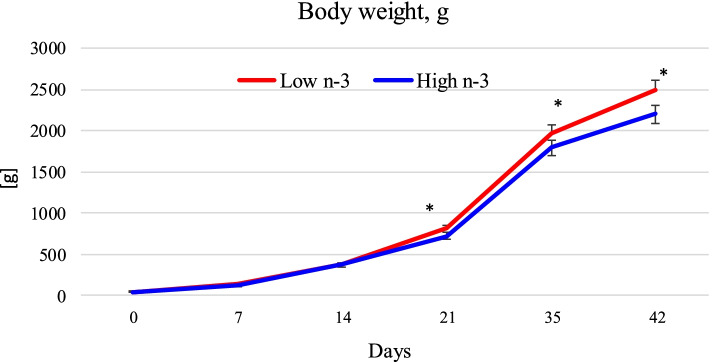
Body weight gain of progeny chicks as affected by maternal diet. High n-3 and Low n-3 represent breeder hen diet containing 3.5% fish oil or sunflower oil as the source of long chain n-3 fatty acid or n-6 fatty acid (linoleic acid). All the chicks were fed the same diet devoid of long chain n-3 or n-6 fatty acids during the 42-day feeding trial. *Significantly different (*P* < 0.05). *n* = 6

Studies on maternal dietary lipids and hatching egg PUFA composition has been well investigated for quality aspects (e.g. weight, hatchability). However, information on post hatch progeny performance are limited. Among the reported studies (summarized in Table 2), most were conducted by feeding an isonitrogenous and isocaloric diets containing 1–5% flaxseed or linseed oil rich in α-linolenic acid, 1–4% fish oil rich in EPA and DHA. Results obtained from such studies were compared to those hens fed diets rich in n-6 fatty or saturated acids such as corn oil, sunflower oil or other lipid sources such as restaurant grease or tallow. In general, with respect to egg quality and hatchability aspects incorporating fish oil in breeder hen diet led to: 1) reduction in egg and yolk weight and, 2) reduced hatchability and chick weight [36, 37]. Over ~ 3 g difference in weight was observed in chicks hatched from hens fed fish oil vs. sunflower oil [38]. The decrease in weight of fish oil chicks persisted during post hatch when raised on a standard diet lacking in long chain n-3 or n-6 fatty acids. At day 42 of growth, chicks hatched from hens fed fish oil-containing diets were 93 g lesser in weight that chicks hatched from breeder hens fed sunflower oil containing diets [38] (Fig. 8). However, not all studies reported such reduction in chick weight. Bullock [37], reported a significant increase in hatched chick and yolk sac weight in chicks hatched from hens fed 3.5% linseed oil (source of α-linolenic acid) compared to chicks from hens fed 3.5% fish oil diets. Overall, these results suggest a significant impact of dietary n-3 fatty acids in breeder hen diet on egg weight, hatched chick weight and progeny post-hatch growth. Some of the discrepancies in results may also be due to differences in breeder hen strain, age or other dietary factors (e.g. other essential fatty acids in diets) [20, 39–41]. For example, addition of fish oil led to a reduction in linoleic acid content of the diet and egg. As an essential fatty acid in poultry, linoleic acid is known to enhance the synthesis of lipoproteins that are needed for egg lipid deposition leading to an increase in egg and yolk weight [41]. Future research using diets with balanced levels of linoleic acid and varying levels of n-3 fatty acid may provide an insight into the role of linoleic acid and n-3 fatty acid in maintaining egg and chick weight in fish oil-fed hens.

**Table 2:** Maternal n-3 fatty acid rich-diet and chick performance post-hatch

Dietary fat source, level and reference	Reported outcome
Soybean or fish oil (55 g/kg) [36]	Inclusion of fish oil led to increased embryonic mortality, reduced hatchability and hatched chick weight.
Fish, flax or safflower oil (3.5%, wt/wt) [37]	Feeding fish oil led to reduction in hatched chick weight.
Fish or sunflower oil (3.5%) or a mix of both oils [38]	Reduction in body weight at day of hatch for chicks from hens fed 3.5% fish oil diet. The differences in body weight observed at hatch persisted during growth and the final body weight was lower in chicks from hens fed fish oil diet. No effect on fertility or hatchability.
Sunflower or fish oil (50 g/kg), or a mix of both [39]	Reduction in day old chick weight by inclusion of fish oil. Effect of maternal diet on post hatch growth persisted up to 3 weeks. At d 42, no difference in body weight or overall feed conversion ratio.
Sunflower, fish, or a mixture of sunflower and fish oil (5%, wt/wt) [40]	No effect on body weight gain and feed efficiency of the broilers during post-hatch.
Fish oil supplementation to obtain EPA:DHA ratio (1/1, 1/2, 2/1) [41]	Lower body weight up to 28-day post hatch. Lower daily weight gain and high feed conversion ratio. Did not improve post hatch performance of chicks.

## Conclusions

In conclusion, n-3 fatty acid nutrition through in ovo sources plays a crucial role in phospholipid PUFA remodeling, lipid metabolism, development and growth and of chicks. Adverse n-3 PUFA supply during embryo development alters tissue phospholipid PUFA molecular species orientation with the potential to disrupt cellular environment, structure, and functions in progeny chicks. From a dietary standpoint, requirements of essential fatty acids in poultry are based on linoleic acid, an n-6 fatty acid. Given the fact that 21-day incubational period contributes to over > 30% of life span in modern day broiler chicks, provision of health-promoting n-3 PUFA highlights the role of early nutrition through in ovo sources. However, the current breeder hen feeding guidelines fail to meet the need of these essential nutrients during early life. Exploring the molecular and cellular biological mechanisms associated with in ovo nutrition will expand our knowledge on the importance of early nutrition. This may also lead to implementation of nutritional regimens to reduce early chick mortality and culls while improving breeder hen as well as neonatal chick health, wellness, and productivity in a natural and sustainable manner. Furthermore, understanding the biochemical and nutrigenomic effects of diet perturbation during early life may open new avenues in other animal as well as human nutritional sciences.

## Data Availability

All data generated or analyzed during this study are available from the corresponding author on reasonable request. The author read and approved the final manuscript.

## Abbreviations

PUFA: Polyunsaturated fatty acid
EPA: Eicosapentaenoic acid
DPA: Docosapentaentoic acid
DHA: Docosahexaenoic acid
PC: Hosphatidylcholine
PE: Phosphatidylethanolamine
ESI-MS: Electrospray ionization mass spectrometry
